# Using linkage maps to correct and scaffold *de novo* genome assemblies: methods, challenges, and computational tools

**DOI:** 10.3389/fgene.2015.00220

**Published:** 2015-06-19

**Authors:** Janna L. Fierst

**Affiliations:** Department of Biological Sciences, University of AlabamaTuscaloosa, AL, USA

**Keywords:** next-generation sequencing, draft genome, scaffolds, physical mapping, optical mapping

## Abstract

Modern high-throughput DNA sequencing has made it possible to inexpensively produce genome sequences, but in practice many of these draft genomes are fragmented and incomplete. Genetic linkage maps based on recombination rates between physical markers have been used in biology for over 100 years and a linkage map, when paired with a *de novo* sequencing project, can resolve mis-assemblies and anchor chromosome-scale sequences. Here, I summarize the methodology behind integrating *de novo* assemblies and genetic linkage maps, outline the current challenges, review the available software tools, and discuss new mapping technologies.

## Introduction

*De novo* genome sequences are fueling a scientific revolution. Biologists are in a position to answer questions that were unimaginable 30 years ago, and new technologies and resources are generating new questions. However, many of these draft genomes contain thousands of individual sequences with no information on how these pieces are assembled into chromosomes. This is problematic both for molecular and developmental studies as individual genes may end up fractured and incorrectly annotated (Baker, 2012; Denton et al., 2014) and for evolutionary studies as fragmented sequences lack the genomic context that is necessary to analyze comparative patterns. For example, the analysis of 12 genomes from closely related *Drosophila* species found increased codon bias and rates of adaptive substitution in genes residing on the X chromosome (*Drosophila* 12 Genomes Consortium, 2007). Relying solely on DNA sequencing means there is no way to identify mistakes in the assembled genome sequence and without a high-quality way to evaluate, correct and anchor next-generation assemblies, they are of limited use.

*De novo* sequencing projects can be successfully paired with a linkage map to address these shortcomings (Semagn et al., 2006; Lewin et al., 2009). Millions of genetic markers can be readily produced with high-throughput sequencing (Baird et al., 2008; Elshire et al., 2011; Heffelfinger et al., 2014), although these large-scale datasets present significant statistical and computational challenges. Genetic linkage maps have been used to refine *de novo* assemblies in organisms ranging from the commercial potato (Xu et al., 2011) to the collared flycatcher bird (Kawakami et al., 2014). There are currently few resources on integrating *de novo* assemblies with linkage maps, particularly for researchers without extensive statistical or computational backgrounds. This article is meant to be a primer for a wide range of biologists interested in using these methods. Below, I outline the scientific problems involved in generating *de novo* assemblies and linkage maps, explain how the two can be integrated, summarize existing computational tools, and describe new technologies for generating physical maps.

## Next generation genome assembly

*De novo* genome assembly works by extracting and sequencing small segments of DNA molecules, and piecing these segments back together into **contigs**, contiguous sequences in which every nucleotide is known (i.e., A, C, G, or T), and **scaffolds**, sequences that contain regions with unknown nucleotides (i.e., N). Next-generation sequencing-by-synthesis has shrunk the price of a million bases of sequenced DNA from $2400 (with Sanger chain-termination sequencing; Sanger and Coulson, 1975; Sanger et al., 1977) to <$0.25 (Liu et al., 2012). The reduced cost makes it feasible for individual investigators to undertake genome sequencing projects, but it carries decreases in read length and accuracy. Sanger sequencing produces 400–900 bp sequencing reads with a per-base accuracy of 99.9% compared to 50–300 bp sequencing-by-synthesis reads (although long reads are possible) (Petterson et al., 2009; Quail et al., 2012). Next-generation sequencing has an average per-base accuracy of 99% but this decreases systematically with high and low GC bias (Dohm et al., 2008) and results in reduced sequencing of these regions (Kozarewa et al., 2009; Chen et al., 2013).

Inserting 30–350 kbp lengths of DNA into plasmids to create bacterial artificial chromosomes (BACs) (O'Connor et al., 1989; Shizuy et al., 1992), cosmids (Collins and Hohn, 1978), and fosmids (Kim et al., 1992) reduces the complexity of whole-genome assembly by effectively breaking the problem down into smaller segments. These genome segments are sequenced and assembled individually but the process is time-consuming and expensive. Sanger sequencing of plasmid clones at 10× depth (where each nucleotide is sequenced, on average, 10 times) can adequately represent the 3 Gb, >50% repetitive human genome (Green, 1997; Weber and Myers, 1997). With fewer reads and longer lengths overlap/layout/consensus (OLC) assembly, in which all sequencing reads are compared pair-wise and assembled based on overlap, is feasible (Myers et al., 2000; Batzoglou et al., 2002). Short read lengths require >100× depth and assembling these large, complex datasets requires sophisticated algorithms like de Bruijn graphs (Pevzner et al., 2001), in which sequencing reads are broken down into short segments of length *k* and these *k-mers* connected in large graphs (Pevzner et al., 2001; Zerbino and Birney, 2010).

Even with sophisticated assembly algorithms, short sequencing reads alone can not generate the information that is needed to discriminate genomic repeats and duplications (Pop and Salzberg, 2008; Alkan et al., 2011) or ancestral polyploidy (The International Wheat Genome Sequencing Consortium, 2014; Chapman et al., 2015). Many current genome sequencing projects rely on mate-pair libraries for long-range sampling throughout the genome (for a review of sequencing strategies, see Ekblom and Wolf, 2014). These specialized libraries select 1–15 kb segments of DNA for circular ligation and extract the ligated ends for traditional short-read sequencing. Assembly algorithms build contiguous sequences from short sequencing reads and use the long-range information provided by mate-pairs to construct large scaffolds (Gnerre et al., 2011). The resulting sequences have high per-base accuracy in gene-rich regions of the genome but do not approximate finished genome sequences (Alkan et al., 2011).

**Figure 1:**
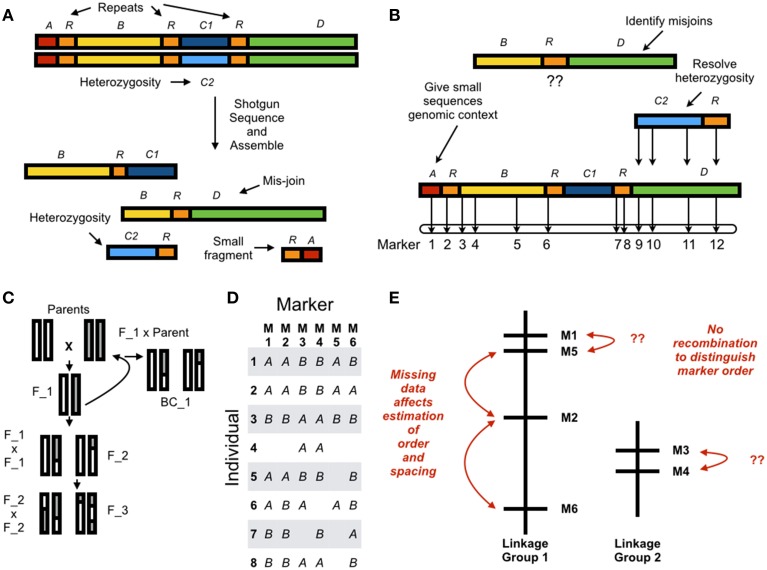
**(A)** In whole genome assembly errors result from residual alleles which appear as discrete sequences in the reference, and mis-joins. Small fragments have no genomic context and contribute little information. **(B)** Using a genetic linkage map to anchor a *de novo* assembly resolves error in the reference sequence by giving small sequences genomic context, resolving allelism, and identifying mis-joins. Chromosome-scale assemblies can be constructed by ordering and orienting sequences with the linkage map. **(C)** A genetic linkage map can be estimated from a parental cross resulting in an F2, F3, or Backcross (here, BC1) population. Estimating a genetic linkage map requires **(D)** genotyping individuals at discrete markers (here, six markers across eight individuals with missing data); and **(E)** grouping markers into linkage groups; and ordering and spacing markers within linkage groups. Estimating order and spacing is difficult due to missing data and little recombination between adjacent markers.

Whole genome shotgun (WGS) assemblies have always suffered from the same limitations, and short read lengths have amplified these problems (Earl et al., 2011; Bradnam et al., 2013; Figure 1A). First, WGS assemblies are inherently fragmented. Eukaryotic genomes contain, at a minimum, millions of nucleotides and “long” contiguous sequences do not approach chromosome-scale. Next-generation assemblies contain many small fragments (on the order of 1000's of nucleotides), and these provide little genetic information. Second, repeat elements are difficult to assemble and can result in mis-joins. Third, diploid individuals, even after extensive inbreeding, will often have residual heterozygosity (Price et al., 2012). These sequences assemble poorly and sometimes occur as duplicated fragments in the assembled sequence. The program REAPR (Hunt et al., 2013) evaluates assembly quality by re-aligning the DNA sequences to the assembled genome but beyond this assessing quality must be done through contig/scaffold length statistics and heuristics combining protein-coding gene annotations with comparative expectations from related species with high-quality assembled sequences (Ekblom and Wolf, 2014). Without a secondary source of information, there is no rigorous way to identify errors.

Longer sequencing reads can build larger contiguous sequences and facilitate higher quality *de novo* assemblies (Alkan et al., 2011) but each of the new platforms has critical shortcomings. Illumina TruSeq synthetic long reads range up to 18,500 bp (McCoy et al., 2014) but rely on parallel library preparation coupled with traditional short read sequencing and bias against assembly of repeats and duplications (Koren and Phillippy, 2015). The Oxford Nanopore MinION passes a single strand of DNA through a protein nanopore (Schneider and Dekker, 2012) and produces reads >20,000 bp but the per-base accuracy is just 70–80% (Quick et al., 2014). Pacific Biosciences single molecule real time (SMRT) sequencing (Eid et al., 2008) produces reads with a median length of 3122 bp but the per-base accuracy is 87% (Koren et al., 2013). Pacific Biosciences long reads can reduce assembly fragmentation when paired with short DNA sequencing reads and this strategy was used to assemble a 128 contig genome sequence for *Drosophila melanogaster* (Landolin et al., 2014). Coupling long-read sequencing with short-read sequencing and assembly will require the development of sophisticated error-correction and assembly algorithms (Koren and Phillippy, 2015).

Moving beyond fragmented genome assemblies requires a linkage map (Lewin et al., 2009; Mascher and Stein, 2014). A high-density linkage map can anchor *de novo* sequences and orient and order small fragments into chromosome-scale sequences (Figure 1B). Inconsistency between markers in the map and markers in the assembled sequences can indicate incorrectly assembled sequences and residual heterozygosity. These can then be resolved to produce a high-quality reference draft genome. For example, the Potato Genome Sequencing Consortium assembled a 727 Mb genome sequence through deep short-read sequencing on Sanger, Illumina, and Roche 454 platforms (Xu et al., 2011). This deep sequencing resulted in an assembled sequence 90% of the estimated genome size and spread across 443 superscaffolds, an impressive but complex and fragmented assembly. Construction of a genetic linkage map yielded 12 linkage groups and 86% of the assembled genome was anchored to these 12 chromosomes.

## Genetic linkage maps

The basic mathematical problem of genetic mapping is: given a set of associations between markers, what is the most likely physical arrangement of these markers on chromosomes? In the early days of mapping these were visible markers like eye color in *Drosophila* (Sturtevant, 1913a,b), at the end of the 20th century these became DNA markers like Restriction Fragment Length Polymorphisms (RFLPs) (Lander and Botstein, 1989), and more recently these have become Single Nucleotide Polymorphism (SNP) markers generated through high-throughput DNA sequencing (for example, Baird et al., 2008; Elshire et al., 2011; Heffelfinger et al., 2014). Constructing a linkage map proceeds in two steps. First, a mapping population must be established to generate recombination and genetic differences between related individuals (Figure 1C). Second, map estimation proceeds by genotyping individuals at different markers (Figure 1D), grouping markers into linkage groups (putative chromosomes), ordering the markers within a group in linear sequence, and spacing the markers according to estimated distances along the chromosome (Figure 1E). Missing data and incorrect marker typing have a large effect on map estimation and infrequent recombination between adjacent markers makes it difficult to order and space markers. These limitations mean that linkage maps are accurate at a large scale but lack fine-scale resolution.

## Current challenges in using linkage maps with *de novo* assemblies

### Establishing a mapping population

Increasing the number of recombination events increases the resolution of the genetic map. This can be achieved by genotyping a very large mapping population but this may be difficult or prohibitively expensive for many organisms. For example, van Oers et al. (2014) constructed a genetic map for the great tit *Parus major* by SNP genotyping over 2000 individuals created from an F_2 cross. For organisms like maize that can be easily bred Recombinant Inbred Lines (RILs) can be established from parental crosses and used for genetic mapping (Burr et al., 1988; Burr and Burr, 1991). However, the necessary time and investment can be prohibitive for long-lived organisms or those that are difficult to breed or grow in the lab. Genetic linkage maps may be estimated from F1 populations (for example, *Eucalyptus grandis* Bartholome et al., 2015) but this requires different algorithms and is not supported by all map estimation software.

### Next-generation sequencing markers

The methodology for estimating linkage maps was originally developed for small-scale data, on the order of hundreds of markers, instead of the millions of genetic markers that are readily produced with high-throughput sequencing (Cheema and Dicks, 2009). The number of possible different orders of genetic markers scales exponentially with the number of markers, and is a major limiting factor in constructing a linkage map. For example, 5 genetic markers in the same linkage group can be ordered in 60 different ways ($\frac{1}{2}$*m*!, where *m* is the number of markers) while 10 genetic markers can be ordered in 1.8 million different ways. The necessary marker density depends on assembly contiguity, and fragmented genome sequences require dense maps for anchoring and orientation. Grouping, ordering and spacing dense marker sets is a central computational challenge and efficient algorithms are still under development (Wu et al., 2008; Strnadova et al., 2014).

Incorrect genotypes and missing data can have a large effect on genetic map estimation, and these problems are magnified by noisy high-throughput SNP genetic markers. Two or more SNPs may be artificially collapsed to a single marker because of sequence similarity in repeats, low-complexity regions, and paralogous genes. Biased sequencing errors may cause one locus to be split into two and uneven sequencing coverage may result from GC bias in polymerase chain reaction (PCR) and sequencing. Sequencing coverage can be uneven across both genomic regions and alleles at one locus due to local GC content. This can result in different data missing from each individual and a negative relationship between sample sizes for markers and individuals.

### No existing software tools to automate the process

Map-assembly integration can proceed in two different ways. For sequence-based mapping genetic markers are aligned to the draft assembly and these markers are used to construct a map, while for array-based mapping the map is constructed first and genetic markers aligned second. For both procedures multiply-mapped markers and loci must be excluded from the final map. However, there is no software to perform either of these processes and it requires custom scripting. Mis-assembled scaffolds can be identified through marker segregation patterns, but in practice identifying and correcting these errors must be done manually. For a typical *de novo* assembly containing thousands of scaffolds and thousands of genetic markers, this quickly becomes time-consuming and subject to error.

## Tools for estimating genetic linkage maps

In Table 1 I summarize software packages for estimating genetic linkage maps that have been used to generate a published map and updated since 2008. Currently there is no single software package that integrates completely with *de novo* assembly, and efficient methods and algorithms are spread across different packages. My goal is to describe the benefits and limitations of each package so biologists can choose which to implement in their own work. For a review of older software, see Cheema and Dicks (2009).

**Table 1:** **Software packages for estimating genetic linkage maps**.

**Package name**	**Strengths**	**Limitations**
**R/qtl** (Broman et al., 2003)	Written in R (user-friendly); High functionality; Integrated graphics; Transparent, open-source implementation; Supported and under current development	Difficulty handling >1000 markers; No methods to address bias in high-throughput DNA sequence markers
**JoinMap** (Stam, 1993; Jansen et al., 2001; van Ooijen, 2011)	User-friendly Graphical User Interface (GUI); Efficient algorithms for grouping and ordering <3000 markers	Only available commercially; Not open-source; Difficulty handling >3000 markers; No methods to address bias in high-throughput DNA sequence markers
**OneMap** (Margarido et al., 2007)	F1 crosses; Written in R; Integrates with R/qtl's functionality and graphics; Transparent, open-source implementation; Robust to genotyping errors and missing data	Difficulty handling >1000 markers; No methods to address bias in high-throughput DNA sequence markers
**MSTMap** (Wu et al., 2008)	Efficient algorithms for linkage grouping and marker ordering; Can handle >10,000 markers	Can not handle F1 crosses; Little documentation; Currently unsupported and may not be under further development; No methods to address bias in high-throughput DNA sequence markers
**Lep-MAP** (Rastas et al., 2013)	F1 crosses; Can handle >10,000 markers; Specialized module utilizes scaffold location of genetic markers in assigning linkage groups	Assumes no recombination in one parent (specialized Lepidopteran mating system; Suomalainen et al., 1973)
**HighMap** (Liu et al., 2014)	Can handle >1000 markers; Utilizes high-throughput sequencing errors in correcting genotyping errors and imputing missing data; Graphics and evaluation functions	Recently published and has not been widely tested

There are several different algorithms for estimating genetic maps (for detailed descriptions of mapping algorithms and performance comparisons see Mollinari et al., 2009; Wu et al., 2011) but these can be generally divided into those that couple iterative marker ordering with probability-based sampling and those that implement graph-based algorithms based on the traveling salesman problem (TSP) (Wu et al., 2008). Under the latter different loci are nodes in a graph and the TSP attempts to connect loci by visiting each node once and only once. The nodes are connected by edges, and the shortest path through the graph is the minimum spanning tree (MST) which approximates the linkage structure underlying the loci. Graph-based algorithms are capable of ordering >10,000 loci (Wu et al., 2008; Rastas et al., 2013). In comparison, marker ordering and sampling algorithms are typically capable of ordering <3000 markers (Margarido et al., 2007; Wu et al., 2008; Cheema and Dicks, 2009; van Ooijen, 2011).

## Developing integrated approaches

Independent genome assembly and map construction can be prohibitively expensive or fail to provide a high-quality assembled sequence for organisms with large, complex, repeat-heavy, polyploid or highly heterozygous genomes. Three published methods (Mascher et al., 2013; Hahn et al., 2014; Nossa et al., 2014) integrate whole genome sequencing with linkage map construction in genome assembly, variant calling, map estimation, and map-assisted assembly to produce assembled genome sequences. PopSeq (Mascher et al., 2013) was used to order 927 Mb of the complex, 5.1 Gb barley genome sequence which is composed of >80% repeats. Recombinant Population Genome Construction (RPGC) was used in a simulated assembly of the 100 Mb genome of the self-fertile hermaphrodite *Caenorhabditis elegans* and produced an assembled genome spread across just 88 scaffolds (Hahn et al., 2014). For a review of these methods, see Mascher and Stein (2014). Nossa et al. (2014) combined *de novo* assembly with linkage mapping to study the organization of the 2.7 Gb genome of the Atlantic horseshoe crab and uncover an ancestral genome duplication.

Genetic linkage maps and *de novo* assemblies have two, complementary scales. Linkage maps are accurate at a large, chromosomal scale, but fine scale marker ordering and spacing are inexact due to infrequent recombination between adjacent markers. In contrast, *de novo* assemblies are accurate at a fine scale (100–1000's of nucleotides) but can not be used to accurately reconstruct chromosome-scale relationships. An integrated approach to *de novo* genome assembly and genetic linkage mapping could utilize the information in each to build a high-quality reference sequence. These methods are just now beginning to appear in computational tools (Liu et al., 2014). For example, LepMap (Rastas et al., 2013) reduces the complexity of linkage group formation with a specialized module that utilizes the scaffold location of genetic markers.

## Physical genome maps

There are several molecular techniques that can generate physical genome maps. Until recently these were prohibitively expensive or difficult to implement but breakthroughs in technology are lowering prices and putting physical maps within reach.

Optical mapping generates ordered, high-resolution maps of restriction sites across single DNA molecules (Schwartz et al., 1993) and can produce high-quality, chromosome-scale physical maps. Optical mapping works by immobilizing single molecules of DNA on a slide, digesting the molecules with restriction enzymes, visualizing the fragments with fluorescence microscopy, and sizing the fragments. The fragments are then pieced together to produce a physical map of the genome with restriction site markers. Optical mapping technology was developed over 20 years ago but its high cost has been prohibitive for most genome projects. Currently, optical maps must still be paired with a high-quality *de novo* assembly but developing nanotechnologies and single molecule sequencing are pushing optical maps to the forefront of genome technology (Levy-Sakin and Ebenstein, 2013). For example, BioNano Genomics Irys System has reduced the price of optical mapping by an order of magnitude and is a feasible platform for studying structural variation in a human genome (Cao et al., 2014).

Hi-C is a molecular technique that cross-links chromatin segments in close physical proximity and quantifies these interactions with high-throughput sequencing (Lieberman-Aiden et al., 2009). The frequencies with which two regions of chromatin interact generates a distribution indicative of the genomic distance between the loci and sufficient for ordering and orienting an assembled genome sequence (Kaplan and Dekker, 2013). The program LACHESIS (Burton et al., 2013) both constructs the frequency-based physical map and aligns scaffolds to the map. Hi-C requires a difficult molecular protocol (de Wit and de Laat, 2012) and has not been widely adopted for genome assembly although it is currently under commercial development and was used to construct genome sequences for a human and the American alligator (Putnam et al., 2015) and *Arabidopsis thaliana* (Xie et al., 2015).

Contiguity preserving transposase sequencing (CPT-seq) (Adey et al., 2014) capitalizes on the unique properties of tagmentation, a recently developed method for both fragmenting DNA and appending sequencing adaptors (Adey et al., 2010). Tagmentation fragments DNA with a Tn5 transposase that binds tightly to target DNA. High molecular weight segments of DNA are extracted and the resulting segments, analogous to a pool of fosmid clones, are sequenced to obtain a phased haplotype (Amini et al., 2014). Combining these phased haplotype segments with an initial genome assembly facilitates the construction of large scaffolds (Adey et al., 2014).

## Conclusions

Coupling *de novo* assembly with linkage mapping is a powerful way to produce a high-quality reference genome. Map estimation was originally developed as a genetic tool over 100 years ago (Sturtevant, 1913a,b) while assembly-specific algorithms and tools are still developing. Linkage maps have proven useful in many different genome assembly projects, and over the next few years assembly-specific algorithms and tools will continue to appear. Physical maps generated with emerging technologies are now becoming feasible for genome sequencing projects.

Dense linkage maps can both orient and order assembled sequences and identify the genetic basis of phenotypic traits. Linkage maps are therefore one of the most important tools we have in genetics. Establishing a mapping population takes time, and undertaking a mapping project is a significant investment of resources. However, linkage maps provide high-quality sequences that can not result from *de novo* assembly alone and every genome project that can reasonably be coupled with a linkage map, should be coupled with a linkage map.

### Conflict of interest statement

The author declares that the research was conducted in the absence of any commercial or financial relationships that could be construed as a potential conflict of interest.
